# A hull reconstruction–reprojection method for pose estimation of free-flying fruit flies

**DOI:** 10.1242/jeb.245853

**Published:** 2023-11-03

**Authors:** Roni Maya, Noam Lerner, Omri Ben-Dov, Arion Pons, Tsevi Beatus

**Affiliations:** ^1^ School of Computer Science and Engineering, The Hebrew University of Jerusalem, Jerusalem 9190401, Israel; ^2^ Institute of Life Sciences, The Hebrew University of Jerusalem, Jerusalem 9190401, Israel; ^3^ Center of Bioengineering, The Hebrew University of Jerusalem, Jerusalem 9190401, Israel

**Keywords:** Biolocomotion, *Drosophila*, Insect flight, Kinematics, Motion capture, Tracking

## Abstract

Understanding the mechanisms of insect flight requires high-quality data of free-flight kinematics, e.g. for comparative studies or genetic screens. Although recent improvements in high-speed videography allow us to acquire large amounts of free-flight data, a significant bottleneck is automatically extracting accurate body and wing kinematics. Here, we present an experimental system and a hull reconstruction–reprojection algorithm for measuring the flight kinematics of fruit flies. The experimental system can automatically record hundreds of flight events per day. Our algorithm resolves a significant portion of the occlusions in this system by a reconstruction–reprojection scheme that integrates information from all cameras. Wing and body kinematics, including wing deformation, are then extracted from the hulls of the wing boundaries and body. This model-free method is fully automatic, accurate and open source, and can be readily adjusted for different camera configurations or insect species.

## Teaser

**Summary:** An experimental system and a pose-estimation algorithm for measuring the flight kinematics of fruit flies are presented. This method is robust, automatic, open source and generalizable to other species.

## INTRODUCTION

Insect flight is a complex and graceful behavior, resulting from the concerted operations of many physiological subsystems and their interaction with complex unsteady flows (Dudley, 2002; Sane, 2003; Taylor and Krapp, 2007; Dickinson and Muijres, 2016). Understanding how these systems combine and conducting comparative studies and genetic screens require high-quality data of free-flight kinematics. Recent improvements in high-speed videography allow us to acquire large amounts of multi-view free-flight data. Yet, a significant bottleneck in this field is automatically extracting accurate body and wing kinematics from these raw data (Provini et al., 2023). To date, an accurate, automatic and open-source tracking method has been unavailable for the insect flight community.

Current tracking methods can be divided into several categories. (1) Manual tracking, where a three-dimensional (3D) model of the insect is manually fitted to individual frames (Fry et al., 2003, 2005; Liu and Sun, 2008; Kassner et al., 2016; Cheng and Sun, 2018; Lyu et al., 2020; Farisenkov et al., 2022), is relatively accurate but extremely laborious. (2) Landmark tracking of feature points on the insect body and wings, which is either automatic or manual (Hedrick, 2008; Walker et al., 2009; Koehler et al., 2012; Nasir and Mat, 2019). Landmarks based on structured light illumination are currently limited to large insects (Wang et al., 2002; Shumway et al., 2020). (3) Model-based optimization methods fit a 3D insect model by projecting it onto the image planes and matching the projections to the data images (Fontaine et al., 2009; Muijres et al., 2017; Bode-Oke and Dong, 2020; Ben-Dov and Beatus, 2022) or by fitting the model to a 3D hull (Kostreski, 2012). Although such an algorithm (Fontaine et al., 2009) can be used for automatic analysis of many flight events (Muijres et al., 2014), it has been demonstrated that obtaining accurate results using this approach requires a 3D model that mimics the insect and its degrees of freedom (DOFs) very faithfully (Ben-Dov and Beatus, 2022). For example, wing deformation (Wehmann et al., 2019) and wing-hinge flexibility challenge the simple modeling of the wing as a rigid plate connected at a fixed hinge point (Ben-Dov and Beatus, 2022). Additionally, the insect legs and their motion might also need to be modeled to achieve accurate tracking. Finally, (4) hull reconstruction methods generate a voxel-based 3D hull of the insect using ray tracing. The hull is segmented into body and wings, from which the insect DOFs are extracted (Ristroph et al., 2009; Kostreski, 2012; Walker et al., 2012; Faruque and Humbert, 2014; Beatus et al., 2015; Whitehead et al., 2015; Bomphrey et al., 2017; Nagesh et al., 2019; Ahmed and Faruque, 2022; Whitehead et al., 2022). These DOFs include the body position and angular orientation, three Euler angles for each wing and, in one case, also wing deformation (Nagesh et al., 2019). This approach assumes only a generic insect morphology and, hence, can potentially handle a wide range of species. However, current implementations of this approach require improvement to make them fully useful for the community. Some of these methods require significant manual input, mainly because they do not handle body–wing and wing–wing occlusions very well. Such occlusions occur, e.g. in fruit flies, when the wings get close to each other at the end of the backstroke (Ristroph et al., 2009; Beatus et al., 2015; Whitehead et al., 2015). These occlusions might limit the applicability of these methods to insects with relatively small stroke amplitudes, such as hoverflies (Walker et al., 2012) and honey bees (Ahmed and Faruque, 2022), or require many high-speed cameras to handle wing–body and wing–leg occlusions, e.g. in mosquitoes (Bomphrey et al., 2017). Importantly, to the best of our knowledge, no method of the above four categories is open source, which makes them practically inaccessible for the insect flight research community.

Here, we present an experimental system and a hull reconstruction–reprojection algorithm for tracking the flight kinematics of *Drosophila melanogaster* fruit flies. We determined the camera configuration by numerically comparing the wing occlusion between several configurations. Our algorithm is based on constructing the wing hull in two steps: we first construct a crude wing hull and reproject it onto the image plane, which allows us to construct new wing images that include information from all cameras. This resolves a significant portion of the occlusions in the system. In the second step, we find the wing boundaries in each reprojected image, and reconstruct the boundaries in 3D. Flight kinematics is then measured from the hulls of the body and wing boundaries, including both wing pose and deformation. This method is automatic, accurate and open source.

## MATERIALS AND METHODS

### Experimental setup and camera configuration analysis

**Fig. 1.**
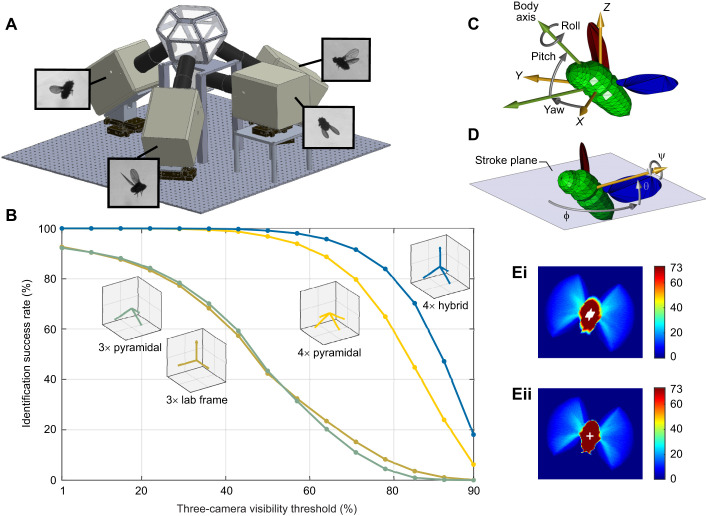
**Experimental system and 2D segmentation.** (A) Experimental setup. (B) Quantifying wing visibility across an ensemble of fly-model poses, used for comparisons between four camera configurations. Three-camera visibility threshold: the minimum percentage of the wing boundary that must be seen in at least three cameras to qualify the identification as successful. (C) Definition of the fly's body axes and degrees of freedom. (D) Definition of the wing Euler angles and stroke plane. (E) Motion-based segmentation of the fly's body. (Ei) When summing the binary images during one wingbeat (73 frames), the most intense pixels correspond to the body. White crosses show the body center of mass (CM) in each summed frame. (Eii) After compensating for body motion, body segmentation and CM estimation improve. Scale on the right of the images in Ei and Eii indicates number of frames.

Fruit flies (*Drosophila melanogaster* Meigen 1831) were placed in a transparent chamber, filmed by four synchronized and calibrated (Theriault et al., 2014) fast cameras (Phantom v2012, Vision Research) with back infrared illumination (Fig. 1A; Supplementary Materials and Methods, Section I). Camera configuration was selected to minimize wing–wing and wing–body occlusions. We computationally quantified the level of occlusions of a given camera configuration by placing a 3D model of the fly (Ben-Dov and Beatus, 2022) in an ensemble of experimentally typical states, and projecting the model to each one of the image planes. For each image, we quantified wing occlusion using two visibility metrics: (1) the percentage of the wing area that is not occluded by either the body or another wing, and (2) the percentage of wing boundary points that are not occluded by either body or wing.

We first compared three single-camera configurations (Fig. S1A): horizontal, vertical and tilted cameras. The visibility score of the vertical camera was the highest, with >95% visibility in both area and boundary-points metrics. The visibility score of the horizontal camera was the lowest, with ∼77%. We then used the boundary-points metric to compare four configurations (Fig. 1B): (1) the commonly used Cartesian three-camera configuration (Ristroph et al., 2009; Zhang and Sun, 2010; Beatus et al., 2015; Muijres et al., 2014, 2017); (2) a three-camera pyramidal orthogonal configuration (Ben-Dov and Beatus, 2022); (3) a four-camera pyramidal configuration (Walker et al., 2012); and (4) a four-camera hybrid configuration, with three pyramidal orthogonal cameras and one vertical (Fig. 1A).

We defined a visibility threshold between 0 and 100% for a successful pose estimation of a given pose (Fig. 1B). A pose is successfully identified if, for each wing, the fraction of boundary points visible by at least three cameras exceeds the visibility threshold. For each threshold value, we calculated the fraction of flight poses across the entire ensemble that are successfully identified. For example, if a threshold of 80% is required, both three-camera configurations achieve successful identification in 3.5% and 7% of the flight poses, while the four-camera configuration succeed in 59% (pyramidal) and 80% (hybrid) of the flight poses. The four-hybrid configuration is consistently superior to the four-pyramidal also at higher values of the visibility threshold and, therefore, was selected.

### Three-dimensional hull reconstruction

Our goals were to estimate the pose of the fly's body and wings described in the standard 12 DOF representation (Fig. 1C,D) and to quantify wing deformation. First, images are converted into binary format using a simple threshold. Each image is segmented into body and wing pixels (i.e. pixels not occluded by the body), using the fact that the wings move much faster than the body (Fig. 1E; Supplementary Materials and Methods, Section II). This motion-based segmentation is more robust than intensity-based methods.

In 3D hull reconstruction of an object, a voxel is set to 1 if and only if its corresponding pixels belong to the object. To reduce the number of calculated voxels, we construct a 3D voxel grid as described in Supplementary Materials and Methods, Section III. To reconstruct a hull from a set of binary images, we project the voxel grid onto each image and test each voxel. Formally, define the projection (*x*_p_,*y*_p_)*_j_*=*f_j_*(*x*_v_,*y*_v_,*z*_v_) from voxel (*x*_v_,*y*_v_,*z*_v_) to a pixel (*x*_p_,*y*_p_)*_j_* on the *j*th camera binary image (Supplementary Materials and Methods, Section I). We use two equivalent notations for a 3D hull. A set notation 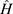:
(1)

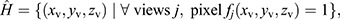
and a functional notation 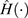:
(2)

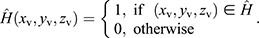


#### Body and wing hulls

**Fig. 2.**
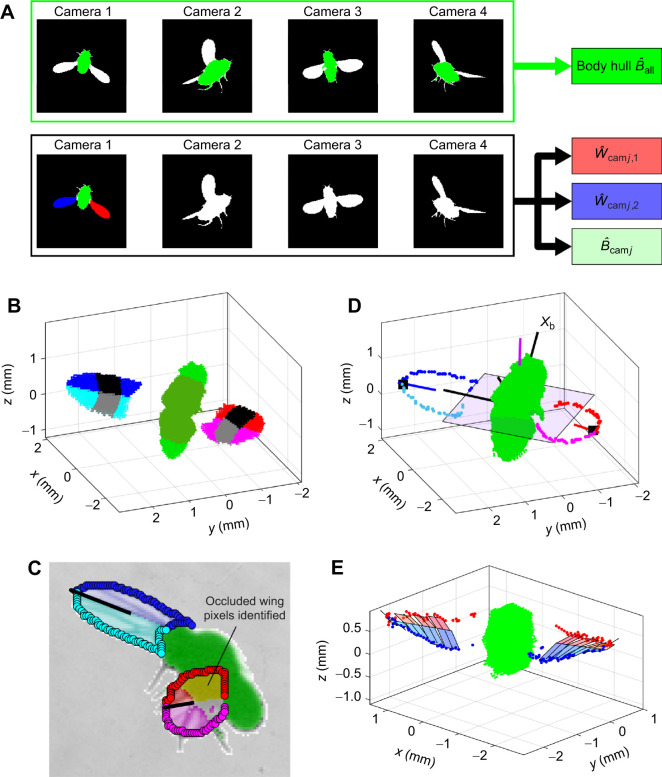
**Pose estimation by hull reconstruction and reprojection.** (A) Combining 2D image to obtain body hull and wing–body expanded hulls. Body hull 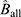 is obtained by reconstructing the body images in all four views. Expanded wing hulls 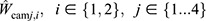 are obtained by reconstructing a wing-only image in one view (here, *j*=1) and the entire fly image in the remaining three views. Expanded body hulls, 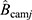 are obtained similarly. (B) The body hull (dark green) with head and tail blobs (light green). Wing hulls are divided into top and bottom halves (blue/cyan, red/magenta), with the stripes to first approximate wing center of mass (CM; black/gray). (C) Reprojection of 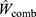 onto the image plains allows us to identify otherwise-occluded pixels. Here, the raw image is superposed with the span vector (black), the identified reprojected wing pixels and their boundaries, divided into leading edge (LE) and trailing edge (TE), as well as the body pixels reprojected from 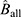. (D) Body hull with the wing-boundary hulls divided into LE (blue/red) and TE (cyan/magenta) voxels. Also shown are the body axes **x**_b_, and the stroke plane (pink) with its normal (magenta). (E) Quantifying wing deformation by calculating five local chord vectors along the wing span, based on the wing boundary voxels (red/blue).

The body hull 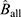 is obtained by reconstructing the four body-only images (Fig. 2A; Supplementary Materials and Methods, Section II). The body center-of-mass (CM) in 3D, **R**_cm,b_, is first approximated as the mean position of the voxels in 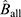, and the body axis **x**_b_ is approximated as the first PCA component of 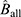. By dividing the body hull into head and tail blobs, we then refine **x**_b_ as the unit vector from tail to head, and **R**_cm,b_ as the midpoint between the blobs (Fig. 2B; Supplementary Materials and Methods, Section IV).

To track the wings it is not enough to reconstruct their hulls from the wing-only images: the resulting hulls are typically partial or even empty when a wing is partially or fully occluded in at least one view. To solve this problem, we build a set of intermediate expanded hulls, by reconstructing a wing/body image in one view together with images of the entire fly in the remaining three views (Fig. 2A). The expanded hulls are then used to identify partially occluded wing voxels, and exclude ‘fake’ voxels that arise from wing–body occlusion.

The expanded hulls are as follows. (1) Eight expanded wing hulls 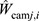 obtained by reconstructing the wing-only image of wing *i*∈{1, 2} in the *j*th camera *j*∈{1...4}, and the images of the entire fly in the other three cameras. If the wings appear as a single connected component in the *j*th view, then 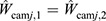. (2) Four expanded body hulls 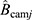 reconstructed from the body-only image in camera *j* and the images of the entire fly in the remaining cameras. (3) Another expanded body hull, 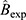, includes only voxels that appear in at least two of the 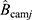 hulls (Fig. 2B):
(3)

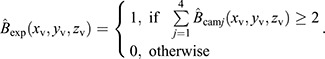


We use the expanded hulls to calculate the combined two-wing hull 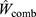, which is the union of the eight expanded wing hulls minus the expanded body hull:
(4)

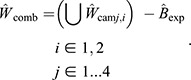


Subtracting 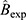 excludes the body voxels as well as many voxels coming from wing–body occlusion. Finally, we separate 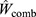 into two wing hulls, 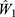 and 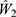, using *k*-means clustering (Fig. 2B; Supplementary Materials and Methods, Section V).

### Wing center of mass, tip, span and chord vectors

For each wing hull 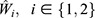, we estimate its CM **R**_cm,w,*i*_, wing-tip position **r**_tip,*i*_, span vector **s***_i_* and chord vector **c***_i_*, in several refinement steps. We first approximate **R**_cm,w,*i*_ as the CM of a voxel strip whose distance from the body **R**_cm,b_ is between 0.40 and 0.65 of the wing length (Fig. 2B). The wing tip is first estimated as the farthest wing voxel from the body CM. The span vector is first estimated as the vector from **R**_cm,w,*i*_ to the tip. Tip estimation is refined by considering the base of a cone of voxels with an axis aligned with the span vector and an apex at **R**_cm,w,*i*_ (Supplementary Materials and Methods, Section V). The span vector is then updated based on **r**_tip,*i*_. To estimate **c***_i_*, we perform *k*-means clustering on the voxels in the above strip within 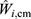 with *k*=2 (Fig. 2B), and approximate **c***_i_* as the unit vector between the CMs of these clusters. Finally, we use these results to improve our selection of the voxel slice and better estimate the wing CM, tip, span and chord (Supplementary Materials and Methods, Section V).

### LE/TE reconstruction and quantifying wing deformation

To 3D-reconstruct the wing leading and trailing edges (LE and TE, respectively), we first reproject the voxels in each wing hull 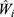 back to the 2D image planes. Importantly, these voxels combine information from multiple views and, therefore, reveal occluded wing pixels (Fig. 2C). The wing-boundary pixels in each of the four reprojected images are then identified as either LE or TE pixels and then projected back to 3D to reconstruct the LE/TE hulls: 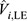 and 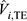 (Fig. 2D; Supplementary Materials and Methods, Section VI).

The fly's wings exhibit some deformation, especially in the TE closer to the body during pronation (Wehmann et al., 2019; Nagesh et al., 2019; Ben-Dov and Beatus, 2022). We use the LE/TE voxels to quantify wing deformation by calculating local chord vectors along the wing span (Nagesh et al., 2019). Additionally, we use the LE/TE to calculate the wing plane and a final estimate for the wing chord **c***_i_* (Fig. 2E; Supplementary Materials and Methods, Section VII).

### Final pose estimation and algorithm validation

Estimating the body **y**_b_ and **z**_b_ axes directly from the body hull is difficult, owing to its nearly cylindrical symmetry (Fontaine et al., 2009). Hence, we estimate **y**_b_ using **x**_b_ and the wing span vectors **s**_1_ and **s**_2_. This is done once per back-stroke, by finding the times when the span vectors are most perpendicular to **x**_b_, averaging these vectors and assigning the result to **y**_b_ (Supplementary Materials and Methods, Section VIII). We estimate **y**_b_ for all times using interpolation, and finally define **z**_b_=**x**_b_×**y**_b_.

The body and wing angles are calculated from the body axes (**x**_b_,**y**_b_,**z**_b_) and the wing span and chord vectors (Fig. 1C,D). The wing Euler angles are defined with respect to the stroke plane. The vector **n**_sp_ normal to the stroke plane is obtained by rotating **x**_b_ by –45 deg in the **y**_b_ axis (Supplementary Materials and Methods, Section IX).

To validate our algorithm, we applied it to an ensemble of synthetic videos generated from 3D fly model with known poses. The standard deviations of the errors in the wing angles were 1.24 deg in φ, 1.43 deg in θ and 2.05 deg in ψ; in the body angles, they were ∼0.5 deg (Supplementary Materials and Methods, Section X).

## RESULTS AND DISCUSSION

**Fig. 3.**
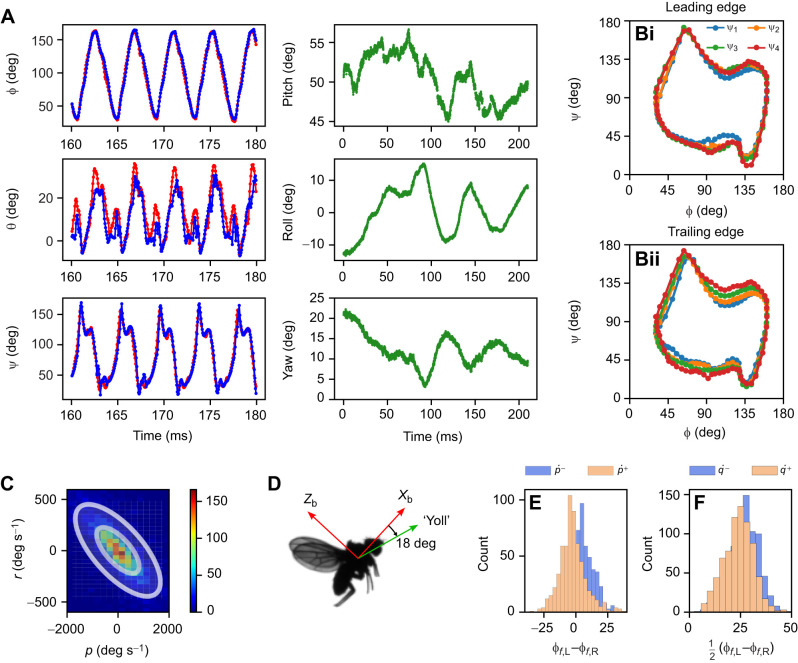
**Pose estimation results.** (A) Sample wing kinematics data for five wingbeats: stroke angle φ, elevation angle θ and the pitch angle ψ, for the left (blue) and right (red) wings. Body angles for the entire flight event are shown in green. (B) Local wing pitch angle in four cross sections ψ_1_…ψ_4_ for the leading (Bi) and trailing (Bii) edges, showing wing deformation. (C) 2D histogram of wingbeat-averaged *r* versus *p*. The negative correlation (fitted Gaussian contours) indicates that the fly's turns are typically coordinated, along the ‘yoll’ axis shown in D. (E) Histograms of the difference between the front stroke angles of both wings (φ_f,L_–φ_f,R_) per wingbeat. Histograms are split by the value of 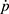. For 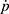
>3×10^5^ deg s^–2^ (orange), the left wing reaches more forward than the right wing and vice versa, indicating that moderate roll acceleration is obtained by front φ asymmetry. (F) Histograms of the mean front stoke angle per wingbeat, split 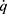. For 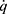
>3×10^5^ deg s^–2^ (orange), the front φ distribution is skewed to the left, indicating that pitch acceleration is achieved by symmetric control of the front stroke angle.

We analyzed 255 flight events with 6706 wingbeats. Sample results of the reconstruction–reprojection process are shown in Movie 1. Fig. 3A,B show unsmoothed sample data from a single event: body and wing angles, including local wing pitch angle due to wing deformation. Fig. 3C,D show various statistics taken over the entire data set. Fig. 3C shows a 2D histogram of *r* (body angular velocity in **z**_b_) versus *p* (body angular velocity in **x**_b_). *r* and *p* are correlated, with *r*∼–(1/3)*p*, indicating that the typical fly's turns are coordinated (Faruque and Humbert, 2014), i.e. combining both yaw and roll, with an effective rotation axis we call ‘yoll’, tilted down with respect to **x**_b_ by tan^–1^(1/3)≈18 deg (Fig. 3D). Fig. 3E shows histograms of the difference between the front stroke angles of both wings φ_f,L_–φ_f,R_ per wingbeat. The data are split according to the wingbeat-averaged body angular acceleration 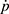. For 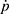
>3×10^5^ deg s^–2^ (associated with roll right), the left wing reaches more forward than the right wing φ_f,L_–φ_f,R_<0, and vice versa. Similar analysis for the backward stoke position does not show significant difference. These results indicate that roll acceleration in moderate turns is achieved mainly by front stroke angle asymmetry. Fig. 3F shows histograms of the mean front stroke angle, split according to 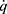, the stroke-averaged angular acceleration in **y**_b_. For 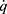
>10^5^ deg s^–2^ (associated with pitch down), the front φ distribution is skewed to the left with respect to wingbeats with 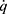
<10^5^ deg s^–2^. This indicates that body pitch acceleration in moderate maneuvers is achieved by symmetric control of the front stroke angle. Both results for roll and pitch were previously demonstrated for small samples of more extreme correction maneuvers (Beatus et al., 2015; Whitehead et al., 2015).

We presented an experimental setup and tracking algorithm for measuring the flight kinematics of fruit flies. The measured kinematics are consistent with the previous literature, and include a new measurement of wing deformation for this species. Camera configuration was selected by minimizing occlusions over several alternatives, yet the algorithm itself is independent of the cameras' number and arrangement. The algorithm operates in several refinement steps and uses a reconstruction–reprojection scheme, which enabled us to identify occluded pixels and extract the wing boundaries in 3D. The method was verified with respect to a synthetic model and demonstrated on a large experimental dataset.

The primary challenge in this pose-estimation problem is wing–body and wing–wing occlusions. Fruit flies represent a particularly relevant test case for this challenge, as their high wingbeat amplitude leads to significant occlusions (e.g. Figs 2C, 3; Movie 2). Adding more camera views increases the amount of information available for pose estimation, but this typically comes at a significant cost. Using our camera configuration analysis tool, it would be possible to assess this trade-off and optimize an imaging setup for tracking other species, for example, insects with an inclined stoke plane such as hoverflies.

The main advance of this pose-estimation method is that it handles occlusions well and, therefore, can robustly and automatically extract the basic body and wing kinematics, as well as wing deformation data. Model-based methods, for example, are highly sensitive to model accuracy (Ben-Dov and Beatus, 2022), and naive hull-reconstruction methods do not handle occlusion very well (Fig. S3, Movie 2). These advances are mainly due to the application of expanded hulls and reprojection techniques, and the optimization of camera configuration.

Because the algorithm is model-free and unrestricted by specific body and wing shapes, it would be relatively straightforward to adjust it for other species with an overall geometry of a body and two wings. Adjusting this method to four-winged species whose ipsilateral wings move as one, such as bees, bumblebees and wasps, would be as straightforward. Additionally, because most occlusions occur when the wings are at the back of the body, this method is expected to perform at least as well for species with lower wing stroke amplitude.

However, this method will work less well on insects with long slender legs, such as mosquitoes. In such insects, even if most leg pixels are removed, the legs still add significant occlusions. Achieving automated tracking for mosquitoes would require either using more camera views (Bomphrey et al., 2017), or employing advanced deep-learning methods (Mathis et al., 2018), for example, by 2D feature extraction followed by geometric triangulation of the features in 3D. Deep-learning method could also be combined with the current hull-reconstruction methods, for example, by providing reliable baseline (Lobato-Rios et al., 2022) and training sets for volume rendering applications (Lombardi et al., 2019 preprint; Mildenhall et al., 2021).

We believe that this open-source algorithm can alleviate the data-analysis bottleneck in the insect flight research community, and facilitate new large-scale comparative studies, e.g. on flight control and the genetic basic of insect flight.

## Supplementary Material

10.1242/jexbio.245853_sup1 Supplementary information
