# Supplementary material for: A hull reconstruction–reprojection method for pose estimation of free-flying fruit flies
Source: J Exp Biol. 2023 Nov 3;226(21):jeb245853. doi: 10.1242/jeb.245853 (PMC10629692; doi:10.1242/jeb.245853)
Supplement: Supplementary information [file jexbio-226-245853-s1.pdf]

Supplementary Materials and Methods

I. EXPERIMENTAL SETUP AND CAMERA CONFIGURATION ANALYSIS

Experimental setup. We used female *D. melanogaster* flies (Canton-S line), 3-5 days old, grown in 25°C and 70% humidity, on standard fly food and under 12-12 hours light cycle. In each experiment, about 20 flies were placed in the transparent flight chamber. The cameras were back-illuminated by near-infrared LEDs (Osram SFH-4780S, λ=810nm), which is invisible for the flies. The vertical camera provides a bottom view though a mirror tilted at 45° (Fig. 1A). The intersected filming volume of the four cameras was $44cm^3$, equivalent to a cube of side length 3.5cm. The cameras were automatically and simultaneously triggered when a fly entered the filming volume, and the resulting movies were saved automatically. Each flight event was ~300ms long, corresponding to ~5,000 frames taken at 16,000 frames per second and 1280×800 pixel resolution. This setup can automatically acquire hundreds of flight events per day.

Camera configuration a nalysis. T his a nalysis, a s well as our entire code, were implemented in Matlab™. We placed the 3D fly m odel i n t he v irtual fi lming vo lume of the tested camera configuration, a nd g enerated a n ensemble of flight poses based on previously measured hovering flight kinematics[1].This ensemble covered the experimentally typical range of flight p oses: body y aw c hanged from − 180° to +170° in increments of 10°, body pitch changed from 15° to 90° in increments of 5°, and body roll changed from −60° to 60° in increments of 10°. Body and wing DOFs are defined in **Figs. 1C,D.** For each combination of the body yaw, pitch and roll angle, we defined 21 wing configurations that span a symmetric wingbeat cycle. Overall, for each camera configuration we considered 157,248 flight poses. For each pose and each camera within a given configuration, we projected the fly model onto the camera plane and obtained its image. For each image, we then quantified w ing o cclusion u sing a n a rea and boundary points metrics. The latter was calculated using 44 uniformly-spaced points on the boundary of each wing.

The projection f_j from a 3D voxel position (x_v, y_v, z_v) in the lab frame-of-reference to a 2D pixel position $(x_p, y_p)_j$ on the j'th image is performed in several steps. First, we calibrate the 4-camera system using the DLT method[2] and obtain the intrinsic and extrinsic camera matrices, including the centers of the cameras in the DLT frame-of-reference. The DLT frame-of-reference is set to be identical to the frame-of-reference of one of the cameras, j_0. To obtain the transformation between the DLT frame and the lab frame, we calculate the rotation matrix, $\mathbf{R}_{\mathrm{DLT}}$, between the z-axis $(0,0,1)$ in the lab frame and the optical axis of the vertical camera in the DLT frame. The origin of the lab frame remains at the origin of the j_0 camera, and the lab-frame x-axis is the rotated x-axis of the vertical camera. This step allows us to convert a 3D point in the lab-

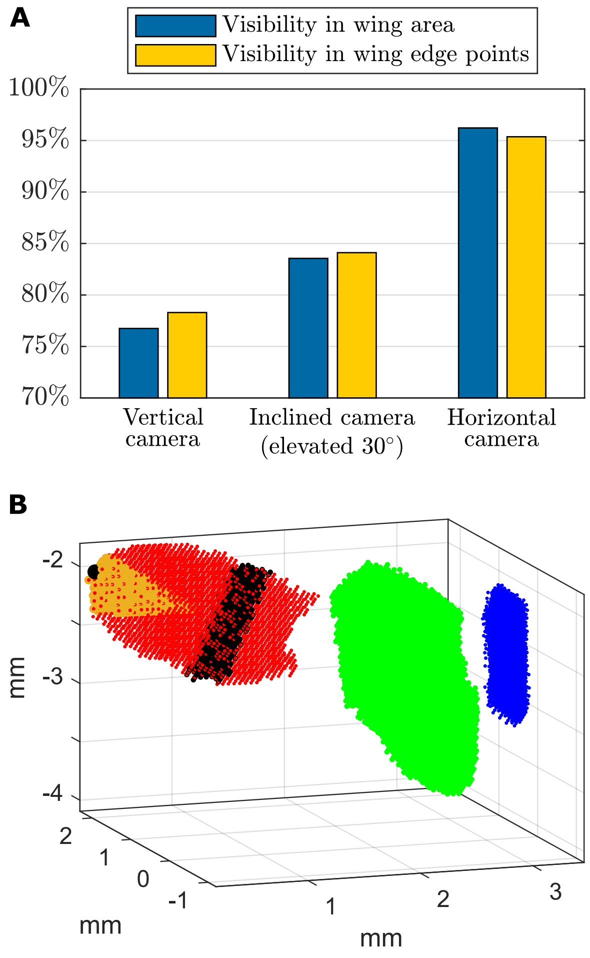

Fig. S1. (A) Comparing single-camera visibility scores. We defined two types of visibility scores: visibility of the wing area (blue bars), and of the wing edge points (yellow bars). The visibility score of three single-camera systems are compared: horizontal, vertical, and tilted cameras. **(B)** Wing tip estimation. The wing tip position (back circle) is refined by finding a cone of voxels (orange), whose apex is at the estimated wing CM and axis is directed along the estimated wing span vector.

Journal of Experimental Biology • Supplementary information

frame to a 3D point in the DLT frame (x_D, y_D, z_D), such that all the 3D reconstruction and reprojection steps are performed in the DLT frame:

$$\begin{bmatrix} x_D \\ y_D \\ z_D \end{bmatrix} = \mathbf{R}_{\mathrm{DLT}} \begin{bmatrix} x_v \\ y_v \\ z_v \end{bmatrix} \tag{5}$$

To convert a 3D point in the DLT frame to a pixel $(x_\mathrm{p}, y_\mathrm{p})_j$ on the $j'th$ camera, we apply the following transformation:

$$\begin{bmatrix} x_p \\ y_p \\ 1 \end{bmatrix} = \mathbf{P}_j \begin{bmatrix} x_v \\ y_v \\ z_v \\ 1 \end{bmatrix} \tag{6}$$

$$\mathbf{P}_j = \mathbf{K}_j \mathbf{R}_j \begin{bmatrix} 1 & 0 & 0 & c_{x,j} \\ 0 & 1 & 0 & c_{y,j} \\ 0 & 0 & 1 & c_{z,j} \end{bmatrix}$$

where $\mathbf{K}_j$ is the 3×3 intrinsic camera matrix, $\mathbf{R}_j$ is a 3×3 rotation matrix from the j_0'th to the j'th camera, and $(c_{x,j}, c_{y,j}, c_{z,j})$ are the coordinates of the j'th camera center in the DLT frame-of-reference. The resulting $\mathbf{P}_j$ matrix is 3×4.

To triangulate a point in 3D based on several 2D image pixels, we perform the inverse transformation using $\mathbf{P}_j^{-1}$, the pseudo-inverse of $\mathbf{P}_j$, which is 4×3:

$$\begin{bmatrix} v_1 \\ v_2 \\ v_3 \\ v_4 \end{bmatrix} = \mathbf{P}_j^{-1} \begin{bmatrix} x_p \\ y_p \\ 1 \end{bmatrix} - \begin{bmatrix} c_{x,j} \\ c_{y,j} \\ c_{z,j} \\ 1 \end{bmatrix} \tag{7}$$

$$\begin{bmatrix} x_{\mathrm{ray}} \\ y_{\mathrm{ray}} \\ z_{\mathrm{ray}} \end{bmatrix} = \frac{1}{v_4} \begin{bmatrix} v_1 \\ v_2 \\ v_3 \end{bmatrix} \tag{8}$$

Subtracting the j'th camera center results in 4-vector representing a ray from the camera center to the 3D point in the DLT frame of reference. Normalizing with respect to v_4 gives the ray as a vector $(x_{\mathrm{ray}}, y_{\mathrm{ray}}, z_{\mathrm{ray}})^T$ in 3D in the DLT frame. Evaluating the intersection of several such rays in the 3D DLT frame allows us to triangulate points in 3D, for example, estimating the fly's CM based on its 2D CM in several images.

II. SEGMENTATION IN 2D

First, we perform a coarse estimate of the body CM. For each frame t we sum up the frames from $(t-\frac{N}{2})$ to $(t+\frac{N}{2})$, where N=72 is the typical number of frames per wing-beat. Considering only the pixels whose value is N+1 in the summed image, filters out the wings and results in a coarse estimate of the body pixels (**Fig. 1Ei**). We then calculate the CM of these pixels in each image. Second, to refine this estimate, it is required to compensate for the body motion. For each frame t we fit a 2nd order polynomial to each of the x and y (coarse) CM coordinates, using data in the range t±100 frames. The refined (x, y) CM coordinates at t are defined as the values of these fitted polynomials at t. To account for the body motion, for each frame t we consider the N+1 frames in the range $(t \pm \frac{N}{2})$ and using the estimated CM from each frame, shift the fly image such that its CM overlaps with the CM at time t. We then sum the shifted N+1 binary images. In this sum image (**Fig. 1Eii**), the body is *bright* and the wings are smeared. Finally, the pixels whose value is N+1 are defined as the body pixels at frame t, and their CM is the final estimate for the fly's 2D CM (Fig. (**Fig. 1Eii**). The remaining non-body pixels at t are segmented as "wing", *i.e.* wing pixels that are not occluded by the body. These wing pixels may appear as two distinct blobs or as a single blob, *e.g.* when the wings are at the back of the body.

III. VOXEL GRID FOR HULL RECONSTRUCTION

To reduce the number of calculated voxels, we construct a 3D voxel grid in three steps. First, we build a coarse 3D grid with 100μm spacing, shaped as a cube around the fly's estimated CM in 3D. This CM is defined as the 3D point closest to the rays emanating from the 2D CMs in all views. Second, we project all voxels onto the 2D images and keep only voxels whose projections overlap with the fly's binary images in all views. We then find the limits of the remaining voxel coordinates in 3D and use them to generate a dense voxel grid with 50μm spacing. This spacing is equivalent to the size of a pixel in 3D. Third, we project every voxel in the dense grid onto the 2D image, and keep only voxels that overlap with the 2D image. This process is computationally more efficient than directly resampling the coarse voxel grid in 3D.

IV. REFINED BODY AXIS AND CENTER-OF-MASS

A first approximation to the body $\mathbf{R}_{\mathrm{cm}}^{\mathrm{b}}$ is the center of mass of the body hull voxels $\hat{B}_{\mathrm{all}}$. To find the $\mathbf{x}_\mathrm{b}$ axis, we first approximate it as the first PCA component of $\hat{B}_{\mathrm{all}}$, labeled $\mathbf{P}_1$, setting the 'head' direction such that $\mathbf{P}_1$ is pointing upwards, *i.e.*, $\mathbf{P}_1 \cdot \mathbf{z}_{\mathrm{lab}}>0$. We then refine the body axis calculation by dividing $\hat{B}_{\mathrm{all}}$ into head and tail blobs **(Fig. 2b)**. First, we find $\mathbf{r}^+$, the farthest voxel from the approximated $\mathbf{R}_{\mathrm{cm}}^{\mathrm{b}}$ in the $\mathbf{P}_1$ direction (head), and similarly calculate $\mathbf{r}^-$ for the $-\mathbf{P}_1$ direction (tail). The estimated body length is the distance $|\mathbf{r}^+ - \mathbf{r}^-|$. The head blob consists of $\hat{B}_{\mathrm{all}}$ voxels whose distance from $\mathbf{r}^+$ in the $-\mathbf{P}_1$ direction is smaller than 20% of the estimated body length, and vice versa for the tail blob. Finally, the body axis vector, $\mathbf{x}_\mathrm{b}$, is calculated as the unit vector from the CM of the tail blob to the CM of the head blob. The refined $\mathbf{R}_{\mathrm{cm}}^{\mathrm{b}}$ is the midpoint between the CMs of these two blobs.

V. REFINED ESTIMATION OF THE WING CM, TIP, SPAN AND CHORD VECTORS

To separate the two wings in $\hat{W}_{\mathrm{comb}}$ we apply k-means clustering on the voxels in $\hat{W}_{\mathrm{comb}}$ with k=4. We then assign the 4 clusters into two wing hulls $\hat{W}_1$ and $\hat{W}_2$: the two clusters

Journal of Experimental Biology • Supplementary information

farthest from the fly's CM are assigned as the 'tip' clusters of each wing. Each of the remaining two clusters is grouped with the closer 'tip' cluster. This procedure works also when $\hat{W}_{\mathrm{comb}}$ consists of a single connected component.

For each wing hull W_i, we first approximate its CM, $\mathbf{R}_{\mathrm{cm}}^{\mathrm{w,i}}$, by finding the voxels in W_i whose distance from the body $\mathbf{R}_{\mathrm{cm}}^{\mathrm{b}}$ is between 0.40 and 0.65 of the wing length (43 voxels, a parameter of the algorithm). We label this set of voxels $\hat{W}_{i,\mathrm{cm}}$, **(Fig. 2B)**. The first approximation of $\mathbf{R}_{\mathrm{cm}}^{\mathrm{w,i}}$, is the center of mass of this strip. Since $\mathbf{R}_{\mathrm{cm}}^{\mathrm{w,i}}$ is expected to be in the middle of this range, this procedure reduces the noise coming from the variability in the number of voxels, especially closer to the body. This estimation is further refined as detailed below.

To find the wing span vector, $\mathbf{s}_i$, we estimate the wing tip position $\mathbf{r}_{\mathrm{tip}}^{i}$, using a similar method to the one used for finding the body axis: a crude approximation that is then refined by calculating the centroid of a subset of the object's voxels. We first find the voxel in $\hat{W}_i$ farthest from the fly's CM. The approximated $\mathbf{s}_i$ is the unit vector pointing from $\mathbf{R}_{\mathrm{cm}}^{\mathrm{w,i}}$ to the estimated tip. This approximation is noisy, because it relies on a single-voxel estimation for the tip. To refine this estimation, we define a cone whose apex is at $\mathbf{R}^{\mathrm{w}}{}_{\mathrm{cm}}{}^{,\mathrm{i}}$, its axis is aligned with the estimated $\mathbf{s}_i$, and its aperture is 15° **(Fig. S1B)**. We calcu-late the projected distance of the voxels in this cone along $\mathbf{s}_i$, select the farthest 5% of these voxels, and calculate their CM, which is defined as the tip ritip. The wing span vector si is then estimated as the unit vector from the i'th wing CM, $\mathbf{R}^{\mathrm{w,i}}{}_{\mathrm{cm}}$, to ritip.

To estimate the wing chord vector, $\mathbf{c}_i$, we perform k-means clustering on the voxels in the wing-voxels strip mentioned above (between 0.40 and 0.65 of the wing length) with k=2 **(Fig. 2B)**. These two clusters correspond to the the top and bottom halves of the wing, containing the leading edge (LE) and the trailing edge (TE), respectively. The identity of the two clusters is determined by the projection of their CM in the $\mathbf{x}_{\mathrm{b}}$ direction, such that the LE cluster projection is positive. The first approximation of $\mathbf{c}_i$ is the unit vector from the CM of the TE cluster to the CM of the LE cluster **(Fig. 2B)**.

We further refine our estimations for the wing CM and chord vector by improving the selection of the voxel slice $\hat{W}_{i,\mathrm{cm}}$ and recalculating the tip, span and chord. Reselecting $\hat{W}_{i,\mathrm{cm}}$ is required because it is originally shaped as a spherical shell, which sometimes distorts the estimations of $\mathbf{R}_{\mathrm{cm}}^{\mathrm{w,i}}$ and $\mathbf{c}_i$, especially when part of the wing hull is missing.

To redefine $\hat{W}_{i,\mathrm{cm}}$, we first calculate the projection each of its voxels onto the span vector. We divide the voxels by their projected lengths into 4 equally long spanwise-segments, and count the number of voxels in each segment. We choose the segment with the largest number of voxels N_{ref} as a reference segment, and calculate its chord length C_{ref} as the distance between its farthest voxels perpendicular to the span vector. We redefine $\hat{W}_{i,\mathrm{cm}}$ as the segment that is closest to the body and similar to the reference segment in terms of its number of voxels and local chord length. To this end, the wing hull $\hat{W}_i$ is divided into 8 segments perpendicular to the span vector. For each segment k the number of voxels N_k and chord length C_k are calculated and compared to the reference section. The similarity is defined by two thresholds: $|N_{\mathrm{ref}} - N_k| < 100$ and $|C_{\mathrm{ref}} - C_k| < 2$ voxel lengths. If more than one of the 8 segments satisfies these conditions, the one closest to the root is selected as the redefined $\hat{W}_{i,\mathrm{cm}}$. The final estimate of $\mathbf{R}_{\mathrm{cm}}^{\mathrm{w,i}}$ is the CM of the selected segment. The final estimate of $\mathbf{s}_i$ is the unit vector from $\mathbf{R}^{\mathrm{w}}{}_{\mathrm{cm}}{}^{,\mathrm{i}}$ to the wing tip. The wing chord vector is calculated from the wing boundaries (Supplementary Section VI).

VI. WING BOUNDARY RECONSTRUCTION USING REPROJECTION

We reconstruct the voxels of the wing's leading and trailing edges by identifying the wing's boundaries in 2D and reconstructing each edge in 3D. Importantly, we use the voxels in the $\hat{W}_i$ hulls, which hold information from multiple views, hence reprojecting them back to 2D can reveal occluded wing pixels (**Fig. 2C**). for each wing i, we perform the following steps:

(1) Finding the wing boundary pixels in each camera view j. We reproject the voxels in $\hat{W}_i$ onto the j'th view and obtain an image per each view. We then find the boundary pixels of these binary images. We also reproject the wingtip $\mathbf{r}_{\mathrm{tip}}^{i}$ and span vector $\mathbf{s}_i$ onto each of the four boundary images **(Fig. S4)**. The tip and span define a line on each image, which divides the boundary pixels into two sets **(Fig. 2c)**. The two *images* of the boundary pixels on either side of this line are designated E_{ij}^{1} and E_{ij}^{2}, respectively **(Fig. S4)**. We use the indices 1 and 2 because it is yet unknown which part of the boundary is the LE and which is the TE.

(2) Divide the wing hull into top and bottom parts. Each voxel with coordinates $\mathbf{r}_{\mathrm{vox}} \in W_i$ is assigned to the top part of the wing if $\mathbf{r}_{\mathrm{vox}} \cdot \mathbf{c}_i \geq \mathbf{s}_i \cdot \mathbf{c}_i$. That is, if $\mathbf{r}_{\mathrm{vox}}$ is 'above' the span vector in the chord direction. The complementary condition defines the voxels in the bottom part of the wing. These two sub-hulls are labeled $W_{i,\mathrm{top}}$ and $W_{i,\mathrm{bot}}$ (**Fig. 2b**). For each camera j, we reproject the $W_{i,\mathrm{top}}$ and $W_{i,\mathrm{bot}}$ voxels onto the j'th view (**Fig. 2c**). These reprojections result in two binary images $H_{ij,}^{\mathrm{top}}$ and H_{ij}^{bot}, respectively. Each pixel in the $H_{ij,}^{\mathrm{top}}$ image may originate from more than one voxel in $W_{i,\mathrm{top}}$, and likewise for $H_{ij,}^{\mathrm{bot}}$ and $W_{i,\mathrm{bot}}$. We keep track of these one-to-many pixel-voxel mappings, to save computation time in the subsequent steps.

(3) Intersections in 2D. At this point, we know that the wing's leading edge pixels are contained in the $\{H_{ij}^{\mathrm{top}}, H_{ij}^{\mathrm{bot}}\}$ images, because these images were obtained from the top and bottom parts of the wing hull. However, we do not know yet which of $\{E_{ij}^{1}, E_{ij}^{2}\}$ corresponds to the leading edge and which corresponds to the trailing edge. The first step in resolving this, is intersecting these two image pairs. For each view j we calculate the four possible intersections of the two image pairs

Journal of Experimental Biology • Supplementary information

$\{E_{ij}^1, E_{ij}^2\}$ and $\{H_{ij}^{\text{top}}, H_{ij}^{\text{bot}}\}$. The resulting images are:

$$\begin{aligned} G_{ij}^{1,\text{top}} &= E_{ij}^1 \wedge H_{ij}^{\text{top}} \\ G_{ij}^{2,\text{top}} &= E_{ij}^2 \wedge H_{ij}^{\text{top}} \\ G_{ij}^{1,\text{bot}} &= E_{ij}^1 \wedge H_{ij}^{\text{bot}} \\ G_{ij}^{2,\text{bot}} &= E_{ij}^2 \wedge H_{ij}^{\text{bot}}, \end{aligned} \tag{9}$$

where the $\wedge$ operator represents an element-by-element logical AND operation between two binary images.

(4) Projecting back to 3D. To find the LE and TE voxels we go back to 3D. For each pixel in each G image (Eq. 9), we can easily find the voxel/s that had generated it. This is thanks to keeping the pixel-voxel reprojection mapping. If, for example, the half boundary in E_{ij}^1 is the leading edge, then it implies that the half boundary E_{ij}^2 is the trailing edge. Then, there should be more voxels corresponding to the pixels in $G_{ij}^{1,\text{top}}$ than voxels corresponding to the pixels in $G_{ij}^{2,\text{top}}$. Additionally, there should be more voxels corresponding to the pixels in $G_{ij}^{2,\text{bot}}$ than to the pixels in $G_{ij}^{1,\text{bot}}$. We, therefore, define the function $\mathcal{V}(\cdot)$, whose argument is a binary image and output is the set of voxels corresponding to the pixels in the image. For each of the two options (E_{ij}^1 is LE and E_{ij}^2 is TE, and vice versa), we count the number of voxels:

$$\begin{aligned} N_{ij}^{12} &= \left|\mathcal{V}(G_{ij}^{1,\text{top}})\right| + \left|\mathcal{V}(G_{ij}^{2,\text{bot}})\right| \\ N_{ij}^{21} &= \left|\mathcal{V}(G_{ij}^{1,\text{bot}})\right| + \left|\mathcal{V}(G_{ij}^{2,\text{top}})\right|, \end{aligned} \tag{10}$$

where $|\mathcal{V}(\cdot)|$ designates the number of voxels in the set. Finally, if $N_{ij}^{12} > N_{ij}^{21}$, we conclude that in the j'th view, E_{ij}^1 is the LE and E_{ij}^2 is the TE, and vice versa. Therefore, we assign:

$$\begin{aligned} G_{ij}^{\text{LE}} &= \begin{cases} G_{ij}^{1,\text{top}}, & \text{if } N_{ij}^{12} > N_{ij}^{21} \\ G_{ij}^{2,\text{top}}, & \text{otherwise} \end{cases} \\ G_{ij}^{\text{TE}} &= \begin{cases} G_{ij}^{2,\text{bot}}, & \text{if } N_{ij}^{12} > N_{ij}^{21} \\ G_{ij}^{1,\text{bot}}, & \text{otherwise} \end{cases} \end{aligned} \tag{11}$$

(5) Combine 3D data from all views to find LE/TE voxels. For the leading edge of wing i, we collect all the *voxels* that correspond to the G_{ij}^{LE} images across all views j. These voxels are collected, with repetitions, into the multiset L_i^{LE} using multiset union:

$$\begin{aligned} L_i^{\text{LE}} &= \biguplus_{j=1}^{4} \mathcal{V}(G_{ij}^{\text{LE}}) \\ L_i^{\text{TE}} &= \biguplus_{j=1}^{4} \mathcal{V}(G_{ij}^{\text{TE}}), \end{aligned} \tag{12}$$

and similarly for the trailing edge voxel multiset L_i^{TE}. Finally, we define the LE hull to include the voxels that appear in L_i^{LE} at least 3 times:

$$\hat{V}_{\text{i}}^{\text{LE}}(l,m,n) = \begin{cases} 1, & \text{if } (l,m,n) \text{ appears in } \hat{L}_i^{\text{LE}} \\ & \text{more than 3 times} \\ 0, & \text{otherwise,} \end{cases} \tag{13}$$

and similarly for the trailing edge hull:

$$\hat{V}_{\text{i}}^{\text{TE}}(l,m,n) = \begin{cases} 1, & \text{if } (l,m,n) \text{ appears in } \hat{L}_i^{\text{TE}} \\ & \text{more than 3 times} \\ 0, & \text{otherwise.} \end{cases} \tag{14}$$

As before, (l,m,n) are the indices of the 3D voxel coordinates in the voxel grid. The final boundary voxels are shown in **Fig. 2d**.

VII. WING PLANE AND LOCAL CHORD VECTORS

For each wing i, we divide the wing span into 5 segments **(Fig. 2e)**. We then calculate the projection of each LE voxel coordinate onto the span vector, and bin these voxels according to the span segments. We do the same for the TE voxels.

The fruit fly's wing are not flat, and exhibit some deformation particularly in the TE closer to the body, especially during pronation[3–5]. We use the LE and TE boundary voxels to quantify wing deformation by calculating local chord vectors along the wing span. For each wing i, we divide the wing span into 5 segments **(Fig. 2e)**. We then calculate the projection of each LE voxel coordinate onto the span vector, and bin these voxels according to the span segments. We do the same for the TE voxels. To calculate the wing plane, we exclude the TE voxels in the 2 bins closer to the body, where most wing deformation occurs, and fit a plane to the remaining voxels. The normal to this plane is designated $\mathbf{n}_i$. The final wing chord vector is then $\mathbf{c}_i = \pm\mathbf{s}_i \times \mathbf{n}_i$, where the + sign is for the left wing and − is for the right wing **(Fig. 2e)**. To quantify wing deformation, for each bin b, we calculate the CM of the LE voxels, $\mathbf{r}_b^{\text{LE}}$, and similarly we calculate $\mathbf{r}_b^{\text{TE}}$ for each TE bin **(Fig. 2e)**. We calculate the vector from the wingtip $\mathbf{r}_{\text{tip}}^i$ to each of these points: $\mathbf{v}_b^{\text{LE}} = (\mathbf{r}_b^{\text{LE}} - \mathbf{r}_{\text{tip}}^i)$. We then keep only the part of $\mathbf{v}_b^{\text{LE}}$ perpendicular to $\mathbf{s}_i$. and re-normalize to find the local chord vectors for the LE bins and similarly for the TE bins:

$$\mathbf{c}_{i,b}^{\text{LE}} = \frac{\mathbf{v}_b^{\text{LE}} - (\mathbf{s}_i \cdot \mathbf{v}_b^{\text{LE}})\mathbf{s}_i}{\left|\mathbf{v}_b^{\text{LE}} - (\mathbf{s}_i \cdot \mathbf{v}_b^{\text{LE}})\mathbf{s}_i\right|} \tag{15}$$

$$\mathbf{c}_{i,b}^{\text{TE}} = \frac{\mathbf{v}_b^{\text{TE}} - (\mathbf{s}_i \cdot \mathbf{v}_b^{\text{TE}})\mathbf{s}_i}{\left|\mathbf{v}_b^{\text{TE}} - (\mathbf{s}_i \cdot \mathbf{v}_b^{\text{TE}})\mathbf{s}_i\right|} \tag{16}$$

VIII. ESTIMATING THE $\mathbf{y}_\text{b}$ AXIS

For each frame t we calculate the angle between the vectors $\mathbf{s}_1$ and $\mathbf{s}_2$, and for each back-stroke we find the frame t_0

Journal of Experimental Biology • Supplementary information

in which these two vectors are most anti-parallel. The division into half strokes is done based on the times when the dot products $\mathbf{s}_i \cdot \mathbf{x}_b$ change sign. For each wing, we average the span vectors within the time window $t_0 \pm 4$ frames. Averaging is done by representing each span unit-vector by two Euler angles and averaging each of these angles while removing outliers. We then invert one of the resulting averaged vectors such that both vectors point to the left side of the body. Next, we calculate the average of the two vectors, take the result's component perpendicular to $\mathbf{x}_b$ and normalize it to a unit vector. The resulting unit vector is defined as $\mathbf{y}_b$ at the mid-forward stroke time t_0.

To estimate $\mathbf{y}_b$ for all frames, we interpolate the two Euler angles of the measured $\mathbf{y}_b$ unit vectors, generate a series of interpolated unit vectors, and for each one keep only the part perpendicular to $\mathbf{x}_b$ at each frame. Based on $\mathbf{y}_b$, the wings, labeled $\{1,2\}$ can now be assigned to the left and right wings, and the body $\mathbf{z}_b$ axis for each frame is defined as $\mathbf{z}_b = \mathbf{x}_b \times \mathbf{y}_b$.

IX. CALCULATING BODY AND WING ANGLES

The wing Euler angles are defined with respect to the wing stroke plane, which is defined with respect to the body frame-of-reference (**Fig. 1**). The vector $\mathbf{n}_{sp}$ normal to the stroke-plane is obtained by rotating $\mathbf{x}_b$ by $-45°$ in the $\mathbf{y}_b$ axis. When the fly is in its nominal flight pose (θ_b=45°, ψ_b=0), the stroke plane aligns with the lab xy plane.

The stroke angle of the left wing, ϕ_L, is obtained by projecting both $\mathbf{x}_b$ and the left span vector onto the stroke plane, and calculating the angle between the two projected vectors. The stroke angle of the right wing ϕ_R is calculated similarly. Each stroke angle is 0° when the wing is in its forward-most position. An increase in either ϕ_L or ϕ_R represent backwards wing motion. The wing elevation angle θ is the angle between the span vector and the stroke plane:

$$\begin{aligned}\theta_L &= 90° - \cos^{-1}(\mathbf{s}_L \cdot \mathbf{n}_{sp})\\ \theta_R &= 90° - \cos^{-1}(\mathbf{s}_R \cdot \mathbf{n}_{sp})\end{aligned} \tag{17}$$

The wing pitch angles ψ are calculated for each chord vector, either the one fitted to the entire wing $\mathbf{c}_L, \mathbf{c}_R$ or the local, spanwise chord vectors. For a given chord vector, $\mathbf{c}$, ψ is the angle between $\mathbf{c}$ and the stroke plane. Let $\mathbf{a}_{sp} = \mathbf{n}_{sp} \times \mathbf{s}$ be a unit vector within the stroke plane and perpendicular to the span vector $\mathbf{s}$. Then, we define the $\mathbf{b} = \mathbf{s} \times \mathbf{a}_{sp}$, and use it to calculate the following two projections and the wing pitch angle:

$$x_\psi = \mathbf{c} \cdot \mathbf{a}_{sp} \tag{18}$$

$$y_\psi = \mathbf{c} \cdot \mathbf{b} \tag{19}$$

$$\psi = \tan^{-1}\left(\frac{y_\psi}{x_\psi}\right), \tag{20}$$

where inverse tangent is caluculated using the `atan2` function in Matlab.

The body angles are calculated per frame using the body axes. The body yaw angle ϕ_b and pitch angle θ_b are calculated from $\mathbf{x}_b$ (**Fig. 1C**):

$$\phi_b = \tan^{-1}\left(\frac{x_{b,y}}{x_{b,x}}\right) \tag{21}$$

$$\theta_b = 90° - \cos^{-1}(x_{b,z}), \tag{22}$$

where $x_{b,x} = \mathbf{x}_b \cdot \mathbf{x}_{lab}$ is the x component of $\mathbf{x}_b$, and similarly for $x_{b,y}$ and $x_{b,z}$. To calculate the body roll angle ψ_b, we first calculate the rotation matrix $\mathbf{R}_{zy}$ that rotates the body frame-of-reference $(\mathbf{x}_b, \mathbf{y}_b, \mathbf{z}_b)$ such $\mathbf{x}_b$ aligns with $\mathbf{x}_{lab}$. This rotation consists of a rotation of $-\phi_b$ in the $\mathbf{z}_{lab}$ axis, followed by a rotation of $-\theta_b$ rotated y axis. Then, ψ_b is:

$$\psi_b = \tan^{-1}\left(\frac{(\mathbf{R}_{zy}\mathbf{y}_b)\cdot \mathbf{z}_{lab}}{(\mathbf{R}_{zy}\mathbf{y}_b)\cdot \mathbf{y}_{lab}}\right). \tag{23}$$

That is, the body roll angle is the inverse tangent of the ratio of the z and y components of the rotated $\mathbf{y}_b$ Fig. 1C.

X. VALIDATION

To validate our algorithm, we applied it to a synthetic dataset of 270 movies generated using a 3D fly model[3]. Body pose was constant per movie and spanned an ensemble of states: body yaw changed from 0° to 100° in increments of 20°; body pitch changed from 25° to 65° in increments of 10°; and body roll changed from −20° to 20° in increments of 5°. Symmetric wing kinematics was extracted from manually-validated experimental data. An overlay of the 3D model and the fly's hull is shown in **Fig. S2A**.

The algorithm's error distributions and their standard deviations are shown in **Fig. S2A**. Outlier in the wing angles estimation are defined as points whose median absolute deviation is greater than 3 (red bars in **Fig. S2**. Outliers are found based on the estimated data, not using the ground truth provided by the model. Hence, outliers can be similarly detected for real data. Overall, 3% of the validated frames were identified as outliers in at least one wing angle. Excluding outliers, the typical errors the wing angles ϕ, θ, ψ (**Fig. S2**) is comparable to an error of a single voxel in the wing CM position.

XI. THE EFFECT OF OCCLUSION

To demonstrate the significant e ffect o f b ody-wing and wing-wing occlusions, we calculated the 3D hull of a fruit fly resulting from the images of the full fly in all four views. **Fig. S3** and **Movie 2** show this hull in black dots together with the output of our algorithm in colored dots. In this representative example, the occlusions resulted in a large protrusion of voxels on the fly's t horax, a s w ell a s bulky w ings. O ther flight pose relative to the cameras may result in different occlusion patterns, which greatly complicate pose estimation.

Journal of Experimental Biology • Supplementary information

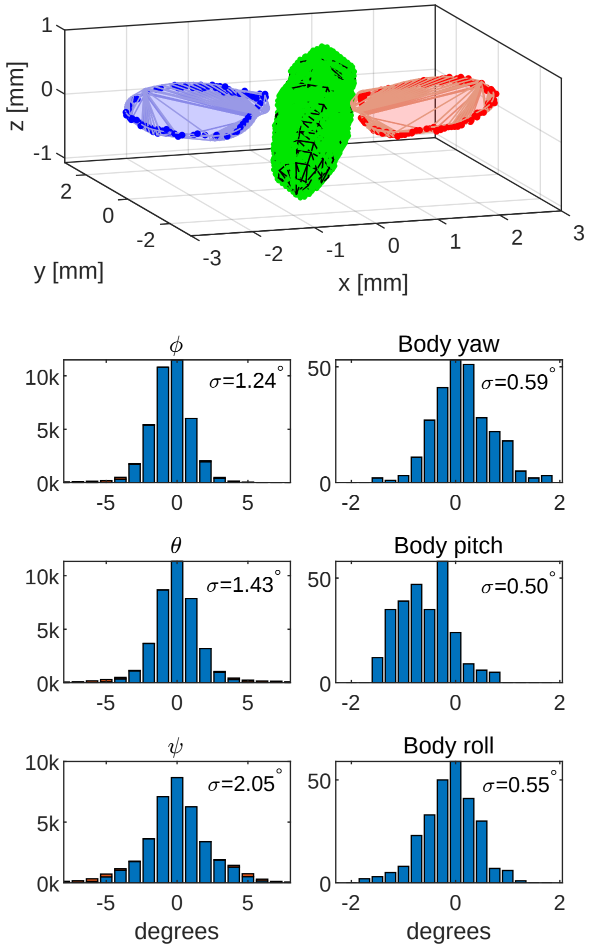

Fig. S2. Validation. Top: the 3D model used to validate our method overlaid with the calculated hulls of the body and wing boundaries. Bottom: The error distribution of the body and wing angles estimated by our algorithm with respect to the ground truth of the model. The red parts of the bars indicate identified outliers. The values of the standard deviations of the errors of the wing angles (ϕ,θ ,ψ) and the body yaw, pitch and roll angles (excluding identified outliers), are shown next to each distribution.

panels on the left show raw footage from the four fast cameras. On top of each image, body pixels are marked in green, the left wing pixels are marked in blue (top half) and cyan (bottom half), and the right wing pixels are marked in red (top half) and magenta (bottom half). Black lines indicate the span vector in each view. Note that wing pixels are identified also when they are occluded by the body. The 3D plot on the right shows the final 3D hull of the fly, with the body in green and the wing leading/trailing edges in the same color code as in the 2D images.

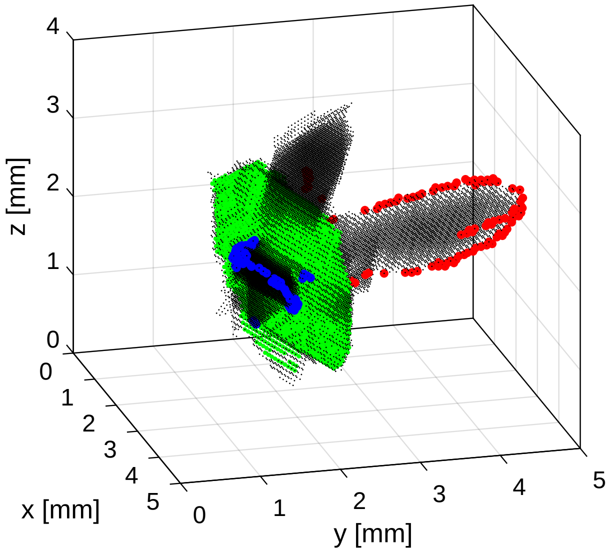

Fig. S3. The effect of occlusion on the 3D hull. Black dots show a typical example of a fly's 3D hull that was calculated from the images of the full fly in all four views. Each dot represents a voxel. The result of our algorithm is shown in colored dots: body voxles (green), right wing boundary (red), left wing boundary (blue). The main effect of occlusion in this example is the large protrusion of voxels on the fly's thorax. Other flight poses might lead to different occlusion patterns. Movie 2 is a 3D representation of this figure.

REFERENCES

[1] T. Beatus, J. Guckenheimer, and I. Cohen, "Controlling roll perturbations in fruit flies," Journal of The Royal Society Interface 12 (2015),397 10.1098/rsif.2015.0075.398.

[2] D. H. Theriault, N. W. Fuller, B. E. Jackson, E. Bluhm, D. Evangelista, Z. Wu, M. Betke, and T. L. Hedrick, "A protocol and calibration method for accurate multi-camera field videography," The Journal of experimental biology 217, 1843–1848 (2014).

[3] O. Ben-Dov and T. Beatus, "Model-based tracking of fruit flies in free flight," Insects 13, 1018 (2022).

[4] H.-N. Wehmann, L. Heepe, S. N. Gorb, T. Engels, and F.-O. Lehmann, "Local deformation and stiffness distribution in fly wings," Biology Open 8, bio038299 (2019).

[5] I. Nagesh, S. M. Walker, and G. K. Taylor, "Motor output and control input in flapping flight: a compact model of the deforming wing kinematics of manoeuvring hoverflies," Journal of the Royal Society Interface 16, 20190435 (2019).

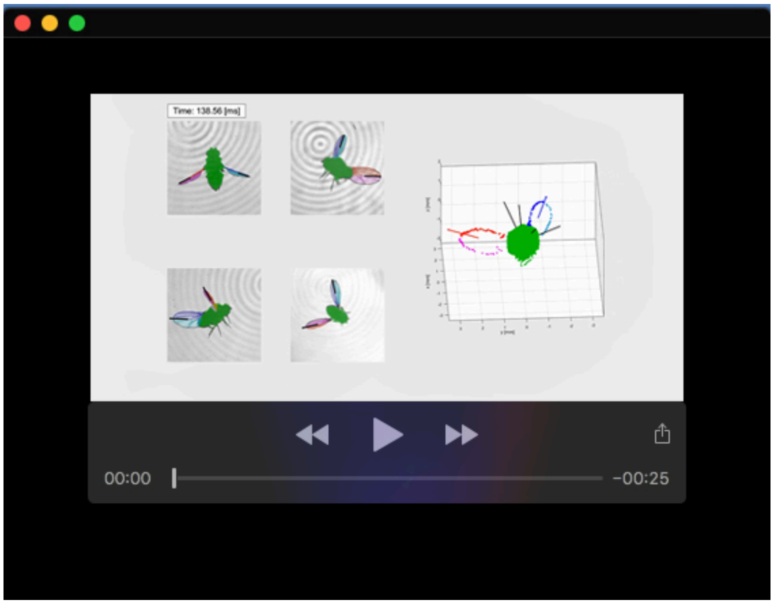

Movie 1. Reconstruction-reprojection results superposed on raw data and shown in 3D for several wingbeats. The four panels on the left show raw footage from the four fast cameras. On top of each image, body pixels are marked in green, the left wing pixels are marked in blue (top half) and cyan (bottom half), and the right wing pixels are marked in red (top half) and magenta (bottom half). Black lines indicate the span vector in each view. Note that wing pixels are identified also when they are occluded by the body. The 3D plot on the right shows the final 3D hull of the fly, with the body in green and the wing leading/trailing edges in the same color code as in the 2D images.

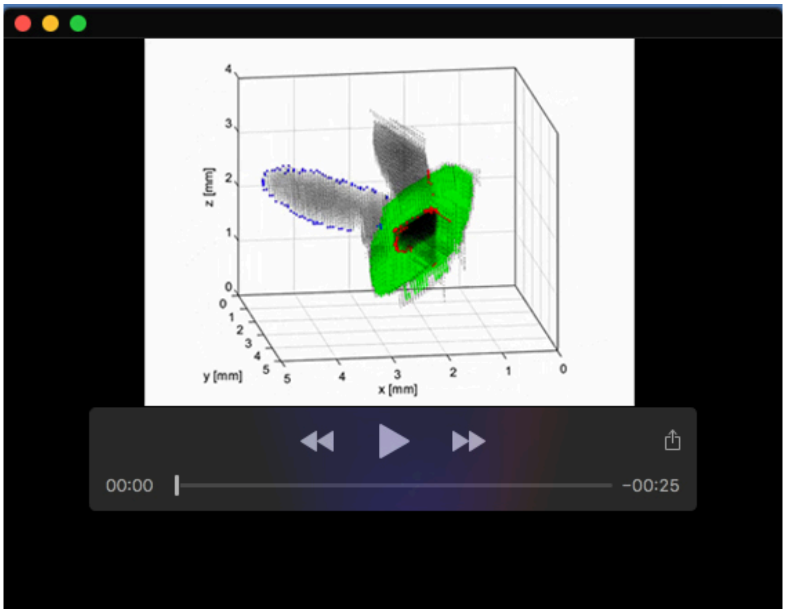

Movie 2. A video version of figure S3, comparing the re-constructed hull using our algorithm vs. a hull generated from the four full-fly views.
